# Evaluation of phrenic nerve stimulation trigger lag and synchronization in different modes of ventilation

**DOI:** 10.3389/fphys.2024.1397070

**Published:** 2024-07-01

**Authors:** Ishmael Bentley, Frank T. Jocewicz, Bruce D. Johnson, Hitesh P. Mehta

**Affiliations:** ^1^ Stimdia Medical Inc., Mendota Heights, MN, United States; ^2^ Mayo Clinic, Rochester, MN, United States; ^3^ Cleveland Clinic, Cleveland, OH, United States

**Keywords:** mechanical ventilation, phrenic nerve, electrical stimulation therapy, diaphragm, atrophy

## Abstract

Phrenic nerve stimulation is currently being investigated for the prevention of diaphragm atrophy in patients with mechanically supported breathing. Patients receiving breathing support from mechanical ventilation are at risk of mismatches between respiratory demand and ventilator support. Our objectives were to determine if a novel phrenic nerve stimulation device provided stimulation during inspiration as intended and did not exacerbate any potential discordances. A benchtop electromechanical simulation model was developed to validate phrenic nerve stimulation with simulated breathing. The phrenic nerve stimulation device was evaluated with a mechanical ventilator attached to a breathing simulator. The trigger ratio and time lag between phrenic nerve stimulation and mechanical ventilation was measured for multiple disease and ventilator parameters. For the 1:1 breath trigger ratio test, 99.79% of intended stimulation breaths received stimulation at the correct time. For the 1:4 breath trigger ratio test, 99.72% of intended stimulation breaths received stimulation at the correct time. For trigger lag times for the inspiratory and expiratory phases, the mean inspiratory lag was 36.10 ± 10.50 ms and 16.61 ± 3.61 ms, respectively. The following discordance scenarios were evaluated in conjunction with simulated phrenic nerve stimulation: asynchrony-false trigger, dyssynchrony-early trigger, dyssynchrony-late trigger, dyssynchrony-early cycling, dyssynchrony-late cycling. Testing demonstrated none of these discordances were exacerbated by the simulated phrenic nerve stimulation. The novel phrenic nerve stimulation device delivered electrical stimulation therapy as intended and did not exacerbate any simulated discordances.

## Introduction

Approximately 800,000 patients in the United States are placed on mechanical ventilation (MV) annually (Rawat et al., 2017). Patients on MV for 21 days or longer have high in-hospital mortality and greater post-discharge mortality, healthcare use, and medical costs compared to those with shorter periods of MV (Hill et al., 2017). Phrenic nerve stimulation (PNS) has been proposed during the weaning phase to prevent atrophy of a patient’s diaphragm to help reduce lengthy mechanical ventilation use (Panelli et al., 2023). A primary concern of phrenic nerve stimulation is inducing or exacerbating patient-ventilator discordances, potentially impacting the patient’s respiratory status. These discordances can be asynchronous or dyssynchronous in nature (Chatburn and Mireles-Cabodevila, 2020). For instance, if the trigger of PNS erroneously happens during a ventilator’s expiration, a sudden ventilator pressure or volume change could cause the ventilator to inappropriately respond.

Stimdia™ Medical Inc. (Mendota Heights, Minnesota) has developed a neurostimulation device, the pdSTIM™ System, for PNS to prevent diaphragm atrophy associated with ventilator-induced diaphragm dysfunction (VIDD) (Schepens et al., 2015). The device is intended to be mechanical ventilator manufacturer and mode agnostic and deliver stimulation to the phrenic nerve during the patient-ventilator inspiratory phase at an operator-set breath rate. A benchtop model was developed to evaluate the ability to deliver PNS at the intended time while not exacerbating any potential discordances.

As a part of pre-clinical testing, a benchtop test set-up was developed to simulate a mechanically ventilated subject. By utilizing this benchtop test set-up, we could simulate a mechanically ventilated subject to measure the performance of the pdSTIM device under various clinical scenarios and multiple ventilator parameter settings.

## Hypothesis and aim

The pdSTIM System is a novel device intended to deliver stimulation to the phrenic nerve on pre-determined inspiratory cycles. The system determines when inspiration begins based on a pre-set inspiratory trigger flow value and delivers stimulation based on its settings. Upon expiration, as determined though a pre-set expiratory trigger flow value, stimulation ceases, until the next pre-set inspiratory trigger is received. It is hypothesized PNS can be delivered via the system at a predetermined time without exacerbating any potential discordances that may be detrimental to the respiratory cycle of a mechanically ventilated patient. The aim of the testing performed was twofold: to evaluate the ability of the pdSTIM device to deliver stimulation during an intended inspiratory cycle while not delivering stimulation during expiration, and to not exacerbate predetermined patient-ventilator discordances (PVDs).

To evaluate whether stimulation delivery occurred as intended, stimulation start and stop response times were evaluated by examining the inspiratory and expiratory lags. The evaluation was performed by examining the delay between the detection of inspiration or expiration by the pdSTIM device and the start or stop of the stimulation signal. Furthermore, the stimulation rate was evaluated by comparing the ratio of stimulated breaths to non-stimulated breaths detected by the pdSTIM device.

The performance of the pdSTIM device during PVDs was evaluated by examining the function of the device during induced discordances by comparing airway flow, airway pressure, and patient effort graphs for those discordances relative to the stimulation signal.

## Materials and methods

**FIGURE 1:**
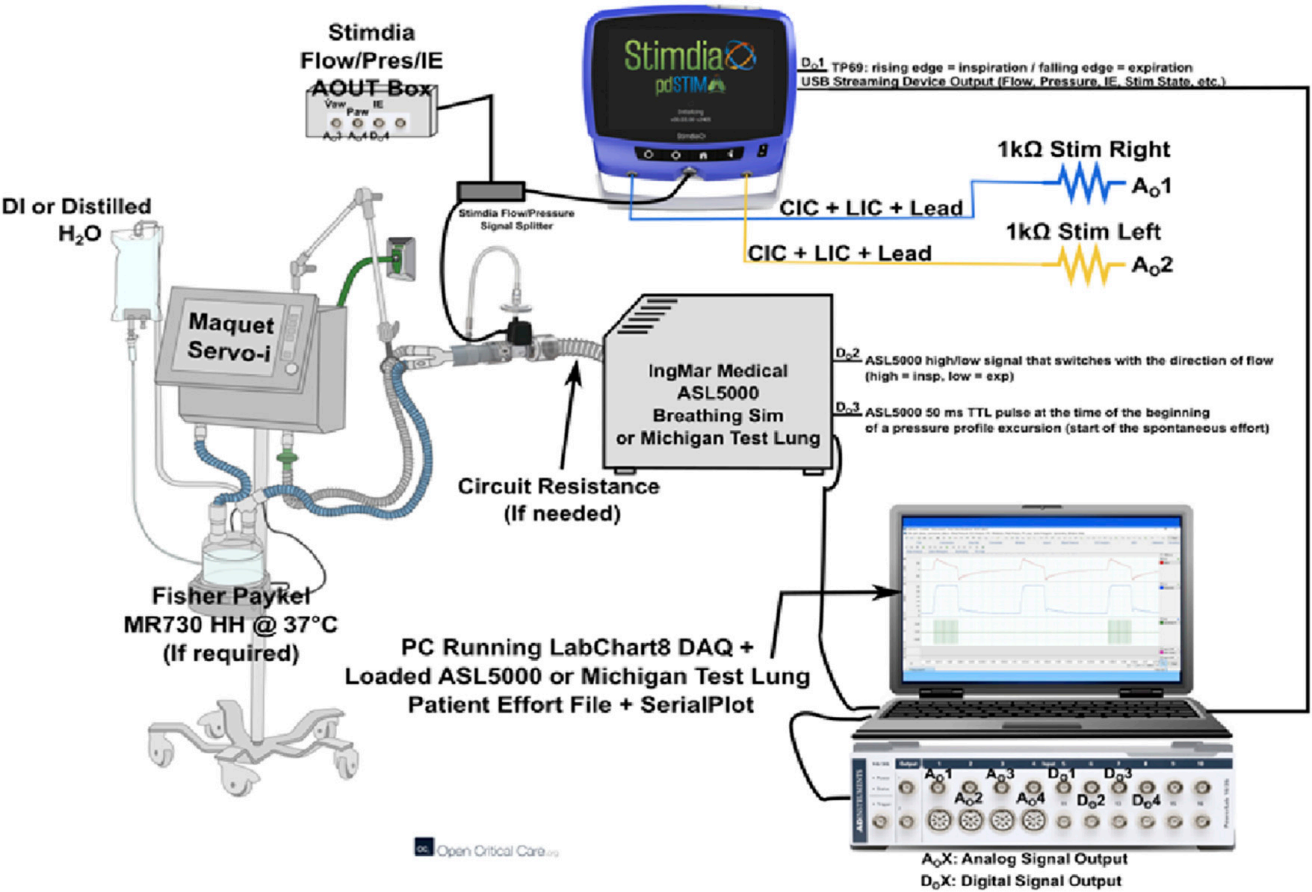
Benchtop set-up.

Testing was conducted by Stimdia personnel at its headquarters in Mendota Heights, Minnesota. The pdSTIM device was used in conjunction with one of two breathing simulators, ASL 5000 (IngMar Medical, Pittsburgh, PA) or DAN (Michigan Instruments, Grand Rapids, MI), and a mechanical ventilator (Maquet Servo-i^®^, Getinge, SE). Connection of the breathing simulator and the mechanical ventilator included standard ventilation circuit tubing and a heated humidifier (MR730, Fisher & Paykel, New Zealand). Included as a part of the ventilation circuit was the Stimdia flow pressure assembly, which includes a component (SFM3300-D, Sensirion, Switzerland) that measures airway flow as well as a component that measures airway pressures (290312000, Nicolay, Germany). A differential probe (Micsig 700 V 100 MHz, Guangdong, China) for stimulation voltage measurement, resistance decade box (380400, Extech, Nashua, NH), and a Windows PC (Redmond, WA) completed the set-up. Data capture and analysis were performed utilizing LabChart (Version 8.1.5, Colorado Springs, CO), MATLAB (Mathworks, Natick, MA), and Python (Version 3.9, Python Software Foundation, Beaverton, OR). Figure 1 below illustrates the entire test set-up.

**FIGURE 2:**
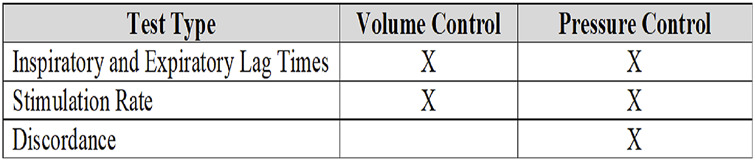
Summary of tests relative to pressure or volume control.

During testing, the Stimdia flow pressure assembly measured flow and pressure values from a simulated breathing circuit. These values are inputs for the pdSTIM device, which allows the device to determine whether the respiratory cycle is in the inspiratory or expiratory phase. For stimulation delivery measurement testing, both volume and pressure control scenarios were examined. For discordance testing, only pressure control ventilation was utilized. Figure 2 below illustrates the type of test performed and whether it was performed in volume or pressure control. Multivariant settings were evaluated to examine the device’s performance in multiple scenarios. Several lung states with varying compliances and resistances were examined, i.e., normal, chronic obstructive pulmonary disease (COPD), and acute respiratory distress syndrome (ARDS). Arnal et al. have classified passively ventilated adult human subjects’ lungs as normal, ARDS, or COPD (Arnal et al., 2018). Normal is classified by compliance of 54 ± 3 mL/cmH_2_O and resistance of 13 ± 3 cmH_2_O/L/s. ARDS is classified by compliance of 39 ± 3 mL/cmH_2_O and resistance of 12 ± 3 cmH_2_O/L/s. Compliance of 59 ± 3 mL/cmH_2_O and resistance of 22 ± 3 cmH_2_O/L/s is classified as COPD. Utilization of multiple-case parameters allowed for device performance to be evaluated under multiple scenarios, with the intent to demonstrate device performance remained unaffected by potential clinical scenarios.

For volume-control tests, tidal volumes of 300 and 800 mL were used. For pressure control, peak inspiratory pressures (above positive end-expiratory pressure [PEEP] values) of 15 and 35 cmH_2_O were utilized. PEEP values of 0 and 20 cmH_2_O were used for all tests. All tests utilized a fraction of inspired oxygen (FiO_2_) value of 21% with an inspiratory:expiratory (I:E) ratio of 1:2 and humidification and temperature of 100% and 37°C, respectively.

Set rates of 12 and 40 Breaths per minute (BPM) were examined, with 12 representing a normal respiratory rate (RR) and 40 representing an accelerated RR. Data for each test run was collected for 40 breaths, resulting in a 60-s test run for an RR of 40 and a 200-s test run for an RR of 12. Regardless of test run-time, all tests recorded data for 40 simulated breaths.

For the tests performed, bipolar stimulation was delivered via two separate electrical leads (right, left) into resistive test loads of 750 and 1000 Ω, respectively. The stimulation pulse train was biphasic, symmetrical, and charge-balanced. The right utilized 100 μs pulse widths at a frequency of 25 Hz with a current amplitude of 15 mA for all pulses. The left utilized 500 μs pulse widths at a frequency of 25 Hz with a ramping current amplitude of 0.5 mA–5 mA at a slope of 5 mA/s.

### Stimulation delivery measurement

The stimulation rate, or the ratio between stimulated and non-stimulated breaths, was controlled via the graphical user interface (GUI) of Stimdia’s device. Stimulation rates of 1:1 and 1:4 were both examined. For volume control, utilizing the information from the scenarios mentioned above, there were a total of 24 test-run scenarios, with each run being executed three times. For pressure control, there were 12 test-run scenarios, with each of these runs performed three times. A total of 108 test runs were conducted for the 1:1 and 1:4 scenarios. For the 1:1 scenario, there was a maximum of 4,320 simulated breaths; for the 1:4 scenario, there was a maximum of 1,080 stimulated breaths, resulting in a maximum of 5,400 combined simulated breaths.

### Discordance evaluation

The following discordances, as defined by Chatburn et al., were examined as a part of the testing (Chatburn and Mireles-Cabodevila, 2020): asynchrony-false trigger, dyssynchrony-early trigger, dyssynchrony-late trigger, dyssynchrony-early cycling, and dyssynchrony-late cycling. Asynchrony is defined as a false or failed trigger or a trigger by another signal other than inspiratory muscle pressure (P_mus,i_). Dyssynchrony encompasses early or late trigger, or cycling and false cycling. Early trigger describes when ventilator-induced pressure (P_vent_) starts before P_mus,i_ (or surrogate), while late trigger describes a clinically significant delay in the start of P_vent_ after P_mus,i_ (or surrogate). A clinically significant advance in P_vent_ return to baseline before P_mus,i_ return to baseline is described as early cycling, whereas a delay is late cycling.

For each discordance scenario, a specific PVD was induced by simulating patient efforts from one of the test lung simulators and modifying the ventilator settings, allowing for analysis of the pdSTIM device’s response during each scenario. The discordances were run in pressure control-continuous spontaneous ventilation (PC-CSV) mode. Each of the PVDs mentioned earlier was tested six times, three for a 1:1 stimulation rate scenario and three for a 1:4 stimulation rate scenario.

### Data analysis

For both stimulation and discordance testing, data analysis was utilized to determine if the pdSTIM System performed as intended. To determine if stimulation was delivered at the appropriate time as intended, i.e., every breath or every fourth breath as determined *a priori*, data was examined and measured against a triggering delay time <88 ms, which is similar to commercially available intensive care unit (ICU) ventilators (Thille et al., 2009). It was considered acceptable if stimulation was delivered when intended, and a test failure if not delivered when intended.

For discordance testing, the airflow and pressure graphs produced from the simulations were utilized in conjunction with the device’s stimulation signal. These were overlaid graphically to examine if the stimulation signal was delivered during the intended breathing phase, i.e., inspiration. It was considered acceptable if the graphical overlay illustrated a stimulation signal that was only active during the intended inspiration cycle.

## Results

**FIGURE 3:**
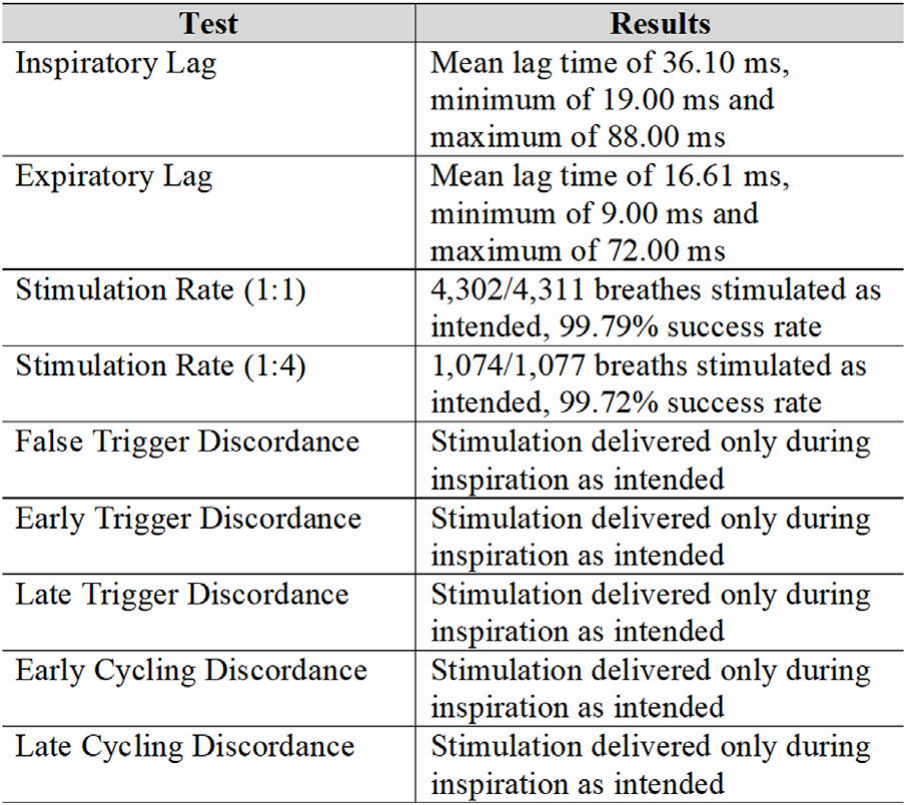
Summary of results.

Figure 3 is a summary of the test results, which includes inspiratory and expiratory lag times, stimulation rate evaluation, and discordance examination. For inspiratory and expiratory lag times, mean values as well as minimum and maximum values are presented. For the two stimulation rates (1:1 and 1:4), the number of total breaths tested and the success rate are presented. For discordances, whether stimulation was only delivered as intended is presented.

### Inspiratory and expiratory lag times

Of the total 5,400 maximum breaths, as explained above, 5,388 breaths were recorded. The mean inspiratory lag was 36.10 ± 10.50 ms with a minimum and maximum of 19.00 ms and 88.00 ms. Hence, all recorded breaths fell at or below the 88.00 ms threshold. The mean expiratory lag was 16.61 ± 3.61 ms with a minimum and maximum of 9.00 ms and 72.00 ms. Based on this data, 95% of the inspiratory lag time values would fall between 15.1 ms and 57.1 ms and 95% of the expiratory lag times would fall between 9.39 ms and 23.83 ms.

For inspiratory and expiratory lag, the trigger lag value of 88 ms was never exceeded. From the data, the mean inspiratory lag of 36.10 ms and mean expiratory lag of 16.61 ms were well below the 88 ms threshold reported in the literature.

### Stimulation rate

For the 1:1 trigger ratio test, 4,311 breaths were simulated, with nine breaths not being stimulated, a success rate of 99.79%. Three failures for the 1:4 trigger ratio test resulted in a success rate of 99.72%. As previously mentioned, the predetermined failure rate was <5%; therefore, the acceptance criteria for each scenario were met. When stimulation was not delivered on an expected breath, it was observed on the next simulated breath. For example, when stimulation was missed in a 1:4 scenario, the system stimulated on the next breath and every fourth breath after that.

### Discordance testing

The graphical figures below provide representative images of the discordance test scenarios. Whereas these figures demonstrate a 1:1 breath ratio, as this was expected to be worst-case, testing was completed for both 1:1 and 1:4. Discordance aside, stimulation only occurred when inspiration was detected in the circuit. The figures also demonstrate that stimulation stopped once expiration was detected.

**FIGURE 4:**
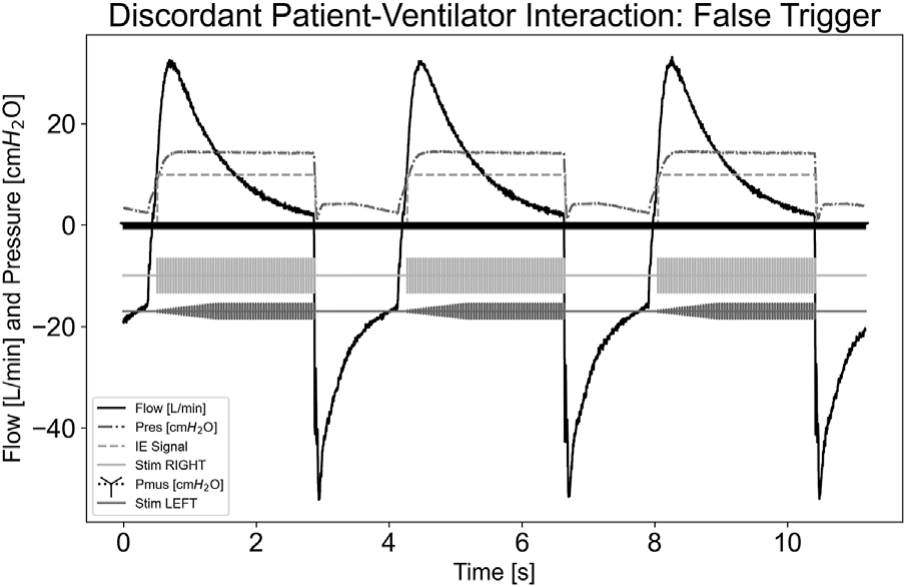
Graph demonstrating stimulation during asynchrony-false trigger patient-ventilator discordance.

In the asynchrony-false trigger scenario, there is no patient effort, as shown in the P_mus,i_ trace equaling 0 cmH_2_O for the test run. A large leak was implemented into the system by opening an access port on the circuit tubing. Though no effort was exhibited, the ventilator still delivered flow. The pdSTIM device stimulated in synchrony with these flow deliveries, as shown in Figure 4. Since the system behaved as expected, this was determined to be acceptable.

**FIGURE 5:**
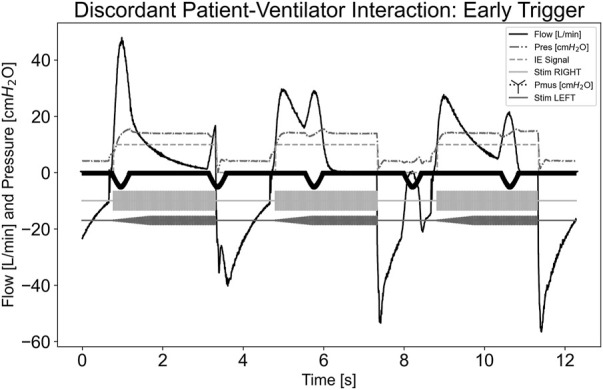
Graph demonstrating stimulation during dyssynchrony-early trigger patient-ventilator discordance.

Figure 5 illustrates a dyssynchrony-early trigger scenario where a breath was delivered without any P_mus,i_ effort, simulating an early trigger event. Since the airway flow was already above the inspiratory trigger and the system state was inspiration, as indicated by the inspiratory-expiratory (IE) signal, stimulation continued until the ventilator cycled into expiration, ending stimulation. The pdSTIM device behaved as expected and was considered acceptable.

**FIGURE 6:**
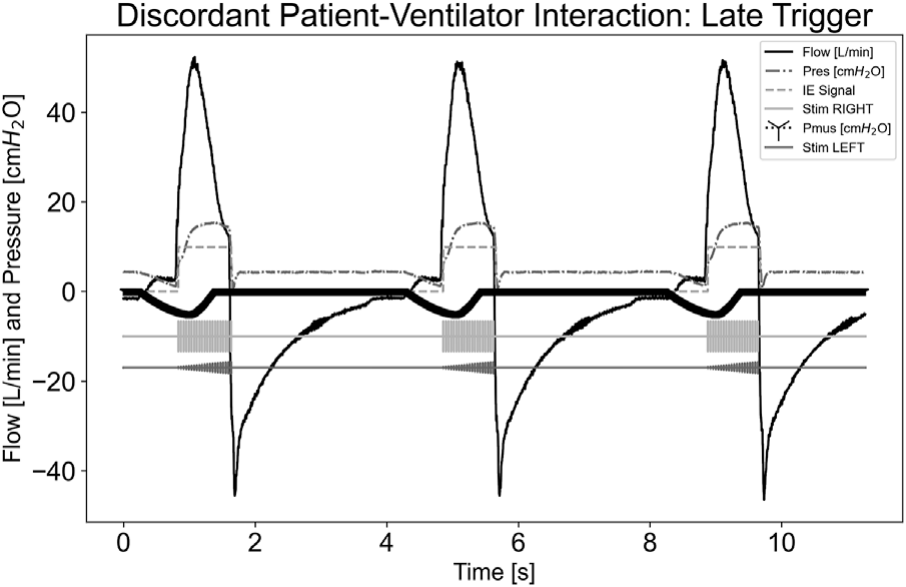
Graph demonstrating stimulation during dyssynchrony-late trigger patient-ventilator discordance.

Figure 6 illustrates a dyssynchrony-late trigger scenario where the ventilator trigger sensitivity was not sensitive enough to detect P_mus,i_ effort in a standard amount of time. Airway flow was not greater than the system’s set inspiratory trigger value; therefore, stimulation did not begin during the initial effort. When the system detected inspiration and began stimulating, this was considered a late trigger. Since the pdSTIM device behaved as expected, this was determined to be acceptable.

**FIGURE 7:**
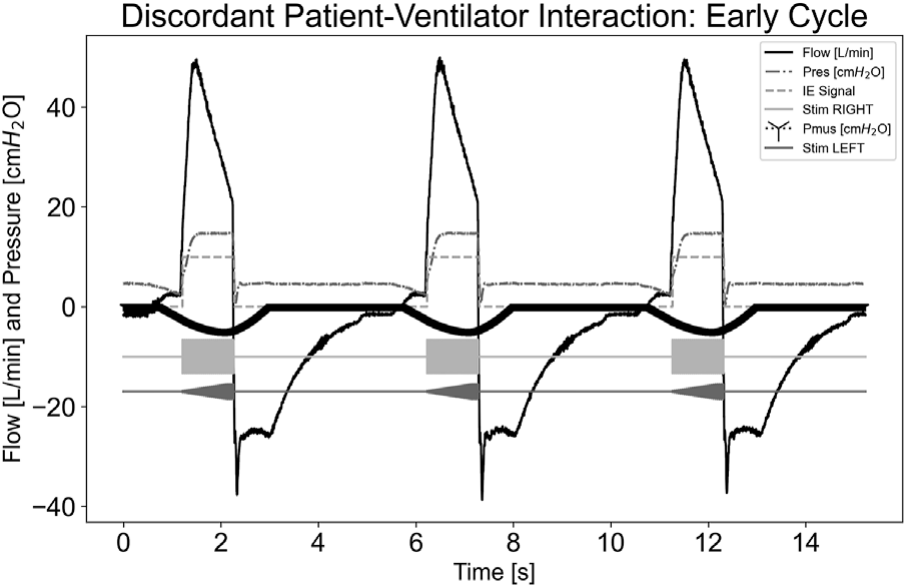
Graph demonstrating stimulation during dyssynchrony-early cycling patient-ventilator discordance.

In Figure 7, a dyssynchrony-early cycling scenario was simulated. As indicated by the P_mus,i_ trace, the simulated effort continued beyond the ventilator cycling. The pdSTIM device maintained synchrony with its detected inspiration and expiration of the measured airway flow. Despite an upward deflection of the flow trace after the ventilator cycled due to simulated effort, the airway flow did not exceed that of the inspiratory trigger of the system; therefore, another stimulation was not started. The pdSTIM device behaved as expected, which was determined to be acceptable.

**FIGURE 8:**
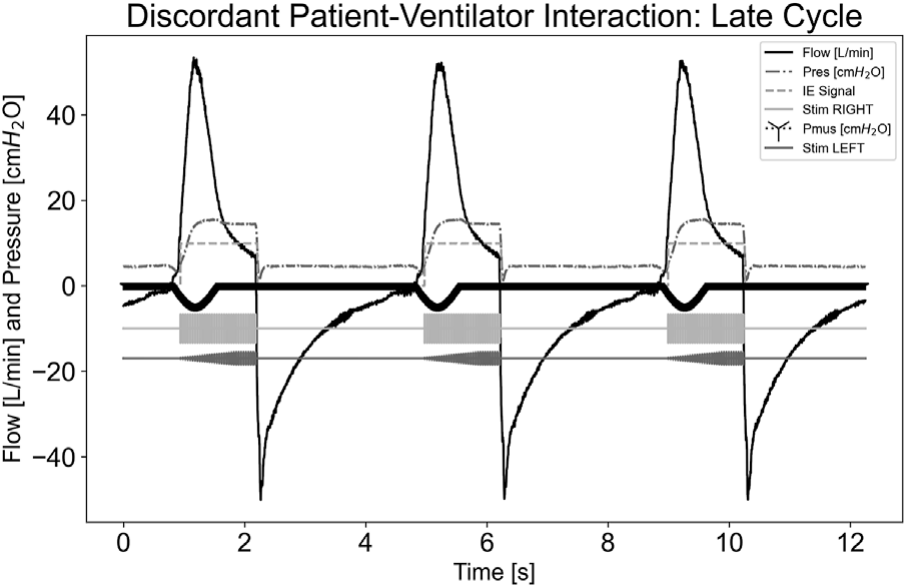
Graph demonstrating stimulation during dyssynchrony-late cycling patient-ventilator discordance.

In Figure 8, a dyssynchrony-late cycling scenario is illustrated, where the simulated effort stopped well before the ventilator cycled. The pdSTIM device maintained synchrony with its detected inspiration and expiration. When the simulated effort stopped, this did not cause the airway flow to fall below the expiratory trigger value. Hence, the pdSTIM device behaved as expected, which is considered acceptable.

## Discussion

Performed testing indicated the pdSTIM device developed by Stimdia Medical Inc. can effectively detect inspiration and expiration and activate or deactivate stimulation accordingly, in a time considered acceptable in the scientific literature. Activation of stimulation with the onset of inspiration, and deactivation of this stimulation during the onset of expiration, is important to simulate the normal function of the diaphragm during breathing. Recognizing inspiration on time and coincidentally delivering electrical stimulation allows for contraction of the diaphragm, exercising it in a manner intended to prevent atrophy of the diaphragmatic muscle. To ensure stimulation is delivered during the majority of the inspiratory phase, it is important to detect inspiration and initiate stimulation at a point not too late in the phase. Concerning expiration, electrical stimulation needs to be deactivated at the onset of expiration to prevent electrical stimulation of the phrenic nerve during this respiratory phase. Electrical stimulation of the diaphragm during what is intended as the expiratory phase would induce a diaphragm contraction, resulting in an unnatural breathing process. Hence, deactivating the pdSTIM device during expiration, as demonstrated in testing, is important to maintain a normal breathing pattern.

Evaluation of the stimulation rate to determine if the pdSTIM device was stimulating during inspiration was important because it determined the device’s ability to provide stimulation therapy as intended. The success rates for the 1:1 and 1:4 scenarios were very high. For the stimulation therapy to perform as intended, the phrenic nerve requires consistent stimulation to allow for activation of the diaphragm in a controlled manner.

Asynchronous or dyssynchronous discordances that may be experienced during mechanical ventilation continue to demand attention from respiratory care professionals. Attention to these potential discordances is important and assumedly will continue to increase in coming years. Any means of phrenic nerve electrical stimulation must demonstrate that it does not cause, contribute to, or exacerbate these potential discordances. As PNS devices enter the clinical study and commercial phases, care must be taken to ensure the devices’ benefits outweigh the risks. From the testing performed and the graphical evaluation of the pdSTIM device under the predetermined discordance scenarios, it appears the pdSTIM device can be used with MV without exacerbating any of the studied discordances. Whereas the clinical impact of the pdSTIM device has yet to be determined, it appears from a discordance safety standpoint the device is low-risk.

### Limitations

With regards to potential limitations of the study, only one mechanical ventilator was utilized as a part of the benchtop set-up. Future iterations of this test may include multiple mechanical ventilators. Furthermore, all potential ventilator settings may be examined. Discordance testing was performed, evaluating one discordance at a time. Future iterations of this may include multiple discordances per test run. For example, instead of only examining dyssynchrony-early trigger in a test run, dyssynchrony-early trigger and dyssynchrony-late trigger may be programmed into one test run.

## Conclusion

The pdSTIM device demonstrated compatibility with multiple disease states, different ventilator modes, and different ranges of ventilator parameter settings and delivered stimulation only during the intended inspiratory phase. The device demonstrated the ability to detect ventilator inspiration and expiration and synchronize stimulation with breath start and stop with mean lag times considerably less than the predetermined 88 ms threshold. The system was demonstrated to deliver stimulation for the intended breaths for 1:1 and 1:4 scenarios. Testing demonstrated the pdSTIM device performed as intended by delivering stimulation during inspiration and ceasing stimulation during expiration. It consistently delivered on the intended breath and did not exacerbate discordances. In summary, the pdSTIM device delivers electrical stimulation at the onset of inspiration on time, stops electrical stimulation in a timely manner, delivers stimulation correctly on the intended breaths, and is low-risk concerning exacerbating discordances.

## Data Availability

The raw data supporting the conclusion of this article will be made available by the authors, without undue reservation.
